# Developmental and functional landscape of theca cells in ovarian biology

**DOI:** 10.1186/s40659-026-00680-3

**Published:** 2026-06-08

**Authors:** Mahnaz Haddadi, Samane Adib, Christiani A. Amorim, Azam Dalman, Mojtaba Rezazadeh Valojerdi

**Affiliations:** 1 Department of Developmental Biology, Faculty of Basic Sciences and Advanced Technologies in Biology, University of Science and Culture, Tehran, Iran; 2 Department of Embryology, Reproductive Biomedicine Research Center, Royan Institute for Reproductive Biomedicine, ACECR, Banihashem Avenue, Resalat Highway, P.O. Box 19395-4644, Tehran, Iran; 3 Department of Anatomical Sciences and Cognitive Neuroscience, Faculty of Medicine, Tehran Medical Sciences, Islamic Azad University, Tehran, Iran; 4 Pôle de Recherche en Physiopathologie de La Reproduction, Institut de Recherche Expérimentale Et Clinique, Université Catholique de Louvain, Brussels, Belgium; 5 Department of Anatomy, Faculty of Medical Sciences, Tarbiat Modares University, Tehran, Iran

**Keywords:** Ovarian theca cells, Theca progenitor cells, Follicular development, Steroidogenesis, Theca cell differentiation, Reproductive endocrinology

## Abstract

**Abstract:**

Ovarian theca cells constitute essential components of the follicular microenvironment and play central roles in follicular development, steroidogenesis, and endocrine regulation. Despite their significance, the developmental origins, differentiation processes, and functional dynamics of theca cells remain incompletely defined, particularly in humans. This review provides an updated synthesis of current knowledge on the ontogeny, molecular signaling pathways, and intercellular interactions of theca cells. It also presents recent findings on the role of theca stem or progenitor cells and their relevance to reproductive disorders, including polycystic ovary syndrome (PCOS), hyperthecosis, and ovarian insufficiency. A comprehensive literature search was conducted using PubMed, Scopus, and Web of Science through May 2025. Search terms included “theca cells,” “theca progenitors,” “follicular development,” “ovarian differentiation,” “steroidogenesis,” and “reproductive disorders.” Original research articles and reviews providing mechanistic insights were included. Current evidence indicates that several signaling pathways, including IGF, SCF, FGF, members of the TGF-β superfamily, GDF9, BMPs, and Hedgehog proteins, are involved in the recruitment, proliferation, and differentiation of theca cells. Bidirectional communication among oocytes, granulosa cells, and theca cells remains essential for folliculogenesis. Increasing evidence links theca cell dysfunction to the pathophysiology of PCOS, primary ovarian insufficiency, and reproductive aging. The identification of theca stem cells (TSCs) and their proposed roles in ovarian regeneration represents an important conceptual advance. A deeper understanding of theca cell biology may guide the development of targeted strategies for infertility and ovarian dysfunction. In contrast, the integration of TSC biology offers new directions for reproductive medicine and reproductive health.

**Graphical abstract:**

**Figure:**
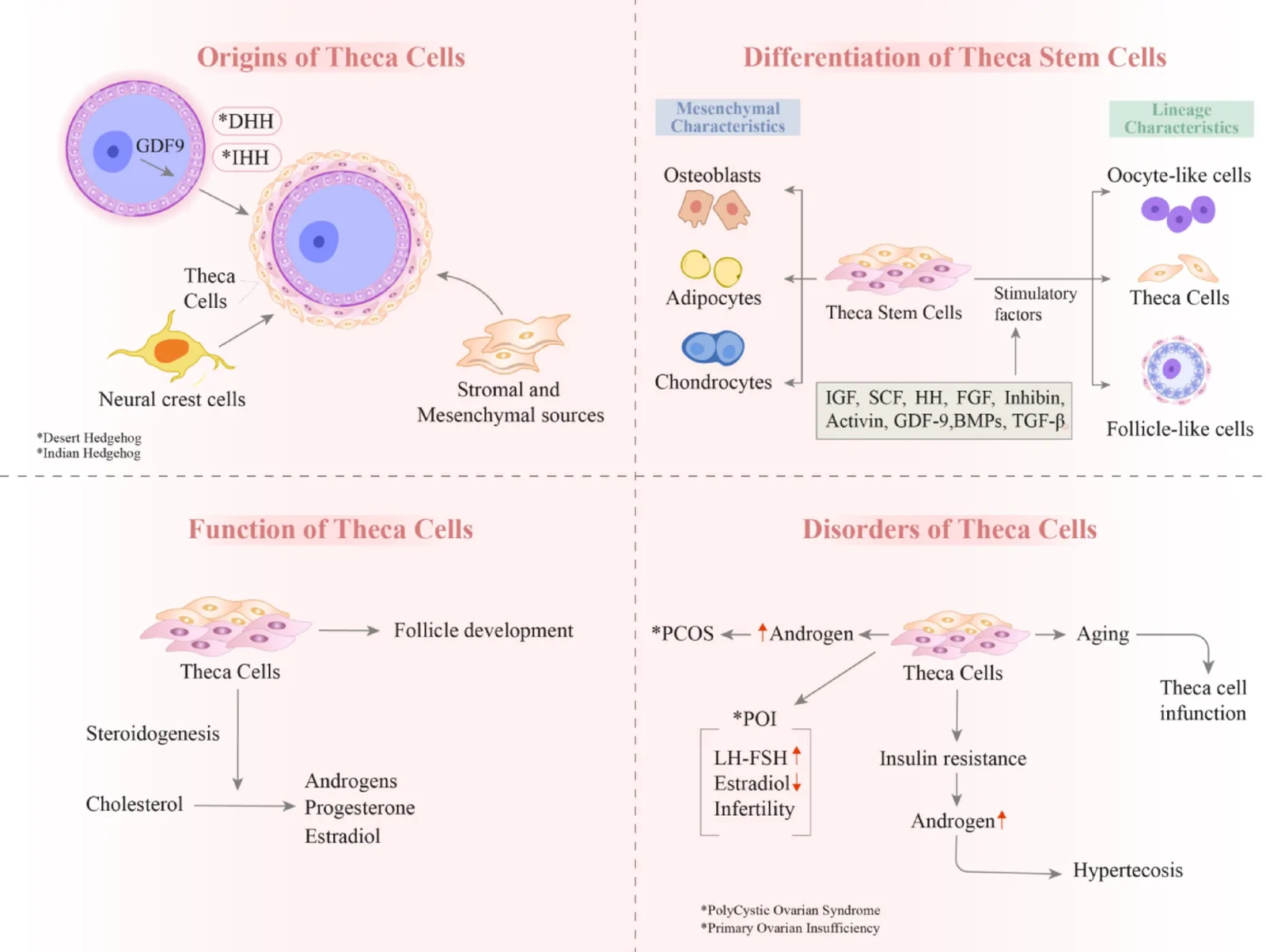

## Background

Theca cells represent a key component of the ovarian follicle and regulate steroidogenesis and follicular development [1, 2]. These cells comprise the theca externa and the theca interna. Theca interna, in coordination with granulosa cells (GCs), produces hormones required for folliculogenesis, ovulation, and normal reproductive function [3]. Despite their central role, theca cells remain less well studied than granulosa cells, leaving significant gaps in our understanding of their origin, differentiation pathways, and interactions within the follicular microenvironment.

Emerging evidence indicates that theca cells perform functions beyond hormone production, including the regulation of follicle maturation and the structural support of the ovarian follicle [4]. Theca cell dysfunction has been associated with major reproductive disorders, including polycystic ovary syndrome (PCOS) [5] and primary ovarian insufficiency (POI) [6], which involve hormonal dysregulation and reduced fertility [5]. These links support the need to expand research on theca cell biology to guide new therapeutic strategies.

Several fundamental questions remain unresolved, including the precise origins of recruited theca cells, the specific functions of fibroblast-like cells within the theca externa, the feasibility of maintaining theca cells in vitro, and the influence of vascular and immune factors on the follicular microenvironment.

This review provides a detailed examination of the origin, recruitment, differentiation, and function of theca cells, with particular attention to their interactions within the follicle and their roles in physiological and pathological conditions.

This review discusses the proposed developmental origins of theca cells across species, including hypotheses that involve stem cell contributions. These hypotheses remain debated, and several studies question the existence and physiological relevance of ovarian stem cells in adult humans [7, 8]. By critically examining both classical and emerging models of theca cell development, this review clarifies the current evidence base and identifies priorities for future research.

### Cellular and structural complexity of the theca layer microenvironment during folliculogenesis

Ovarian follicles represent the functional units of the ovary and consist of the oocyte, GCs, and theca cells. During folliculogenesis, follicles progress through successive developmental stages in a tightly regulated process governed by endocrine and paracrine signaling pathways [9]. At the antral stage, GCs are surrounded by a fully developed theca layer that contains theca cells, vascular endothelial cells, immune cells, fibroblast-like cells, smooth muscle cells, and pericytes. Together, these cell populations shape the structural organization and regulatory processes that govern follicular development [10]. Structurally, theca layer is organized into two distinct regions: theca interna, which is primarily steroidogenic, and theca externa, which provides mechanical support and contains cells involved in extracellular matrix (ECM) production and contractile activity [4].

Theca interna contains steroidogenic theca cells, vascular endothelial cells, immune cells, pericytes, and mesenchymal progenitor cells [4]. Theca interna cells synthesize androgens, including testosterone and dihydrotestosterone [11], which GCs subsequently aromatize to estradiol. In addition to steroid production, theca cells secrete growth factors, including bone morphogenetic proteins (BMPs) and transforming growth factor-β (TGF-β), which regulate granulosa cell proliferation and differentiation [4, 12, 13]. Vascular endothelial cells support angiogenesis and regulate blood flow, and granulosa cell-derived vascular endothelial growth factor (VEGF) promotes these processes [14]. Immune cells, particularly macrophages, increase during follicle enlargement and contribute to immune surveillance, debris clearance, and regulation of follicular atresia [15, 16]. Lymphatic vessels within the theca layer also contribute to immune homeostasis.

**Fig. 1:**
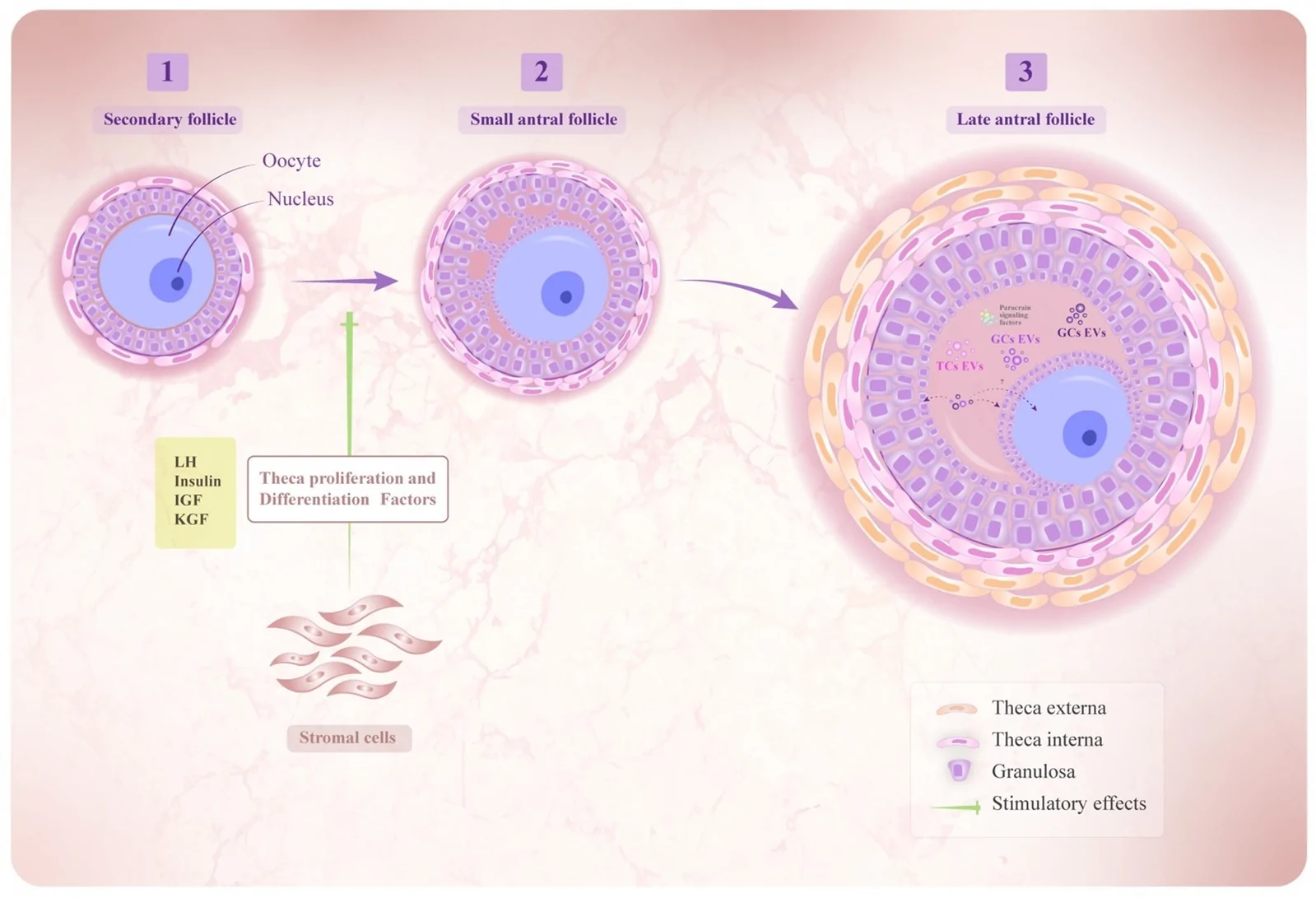
Development of the theca layer during folliculogenesis. The illustration depicts the formation and maturation of theca interna and theca externa during follicle development and oocyte maturation in mammals. From the secondary follicle stage, stromal cells differentiate into TCs in response to stimulatory factors such as LH, insulin, IGF, and KGF. As follicles progress to the small antral and late antral stages, theca interna and theca externa become clearly organised around the granulosa cell layers [18]. Theca cells proliferate and differentiate, contributing to follicular growth through paracrine interactions and the release of EVs. Theca interna cells acquire steroidogenic activity, while theca externa cells provide structural support

In contrast, the theca externa serves as a mechanically active layer that maintains follicular integrity, contributes to ovulation, and provides ECM components [1, 17]. It contains fibroblast-like cells, smooth muscle cells, pericytes, and immune cells that cooperate to sustain vascularization and regulate follicular biomechanics (Fig. 1) [4, 15]. Recent studies in murine ovaries show that contractile theca cells generate compressive forces through actomyosin-dependent mechanisms and fibronectin-mediated ECM assembly, which regulate follicle expansion and influence granulosa cell signaling pathways, including YAP [18]. Disruption of theca contractility or basement membrane integrity alters pressure dynamics, impairs follicle growth, and reduces developmental competence [17].

Consistent with their distinct functions, theca interna cells are enriched in smooth endoplasmic reticulum (SER) and mitochondria to support steroidogenesis, whereas theca externa cells display fibroblast- and smooth muscle-like characteristics associated with ECM synthesis and mechanical regulation [1, 19, 20]. The presence of endothelial cells, immune cells, pericytes, and mesenchymal progenitors indicates that the thecal compartment functions as a dynamic stromal niche rather than a passive boundary [1, 21]. ECM components of the theca layer, including fibronectin (FN1) and type I collagen (COL1A1), provide structural scaffolding and regulate follicular biomechanics [22]. Matrix metalloproteinases such as MMP2 mediate ECM remodelling and support follicular expansion [23, 24], whereas LOX promotes collagen cross-linking and increases ECM stiffness, which influences mechanosignalling and tissue integrity [25]. Dysregulation of these processes disrupts normal follicular maturation and ovulation.

Species-specific features further shape the thecal layer. For example, in Wistar albino rats, fibroblast-like cells in the theca externa synthesize connective tissue, creating a responsive microenvironment that accommodates follicular growth [26]. Across species, the ECM regulates growth factor bioavailability, cell–cell interactions, and vascular tone, highlighting the multifunctional role of smooth muscle cells [27].

**Fig. 2:**
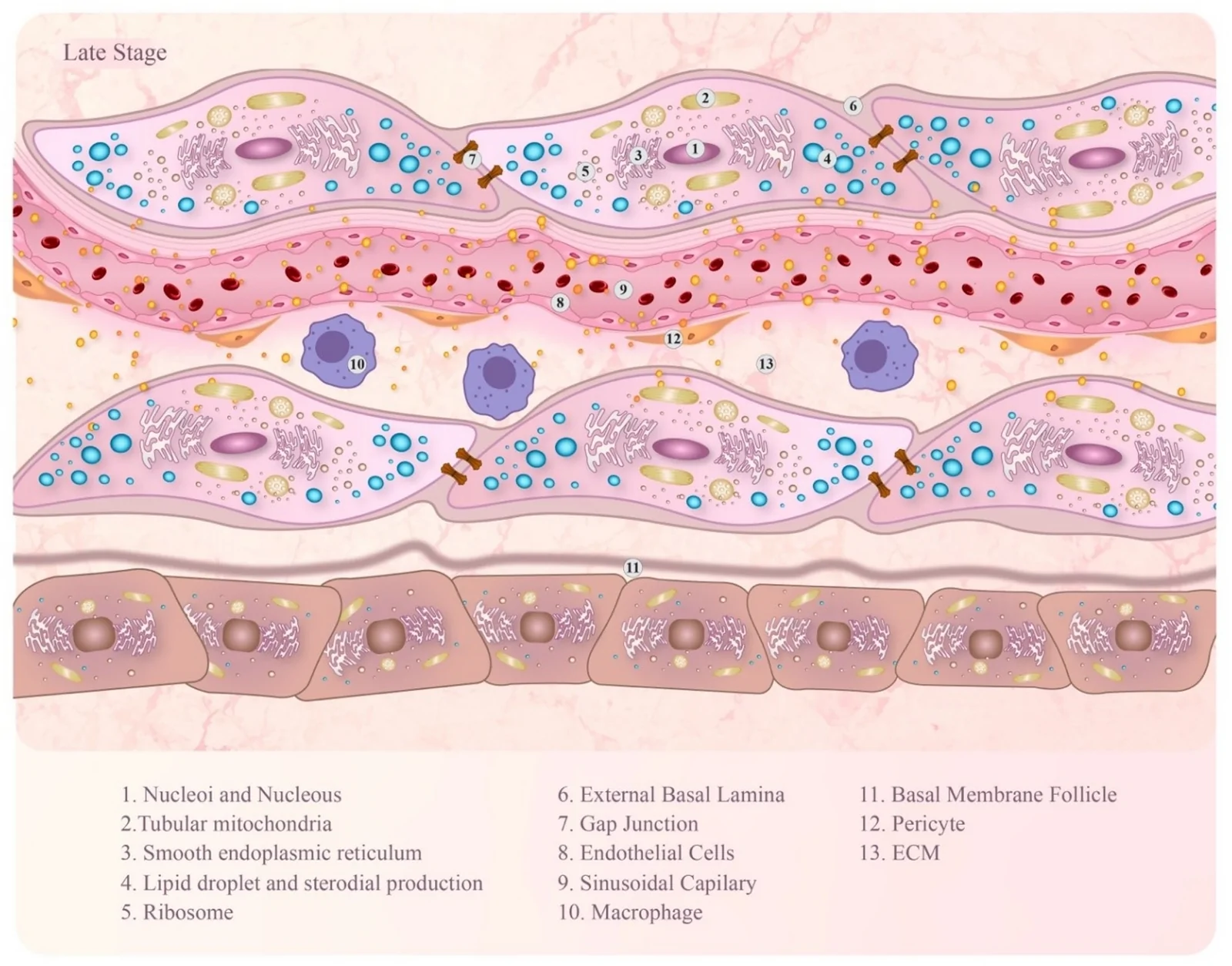
Ultrastructural changes in endocrine theca cells. The illustration depicts the ultrastructure of theca cells during late follicle development. At this stage, differentiating theca cells undergo hypertrophy, become less flattened, extend pseudopodia, and show increased steroidogenic capacity. Lysosomes, lipid droplets associated with steroid production, tubular mitochondria, and ribosomes become more prominent. Theca cells also show non-steroidal secretory activity. These structural changes align with the shift toward active steroidogenesis as folliculogenesis progresses. The ECM and cellular composition of the theca layer support follicular development by providing a structural and biochemical framework for growth and maturation. By integrating hormonal signaling, vascularization, immune defense, and mechanical support, theca compartments contribute to ovarian function and reproductive health

Ultrastructural studies in human ovaries show that, as follicles transition from pre-antral to antral stages, theca interna cells exhibit increased SER volume, greater mitochondrial density, and accumulation of lipid droplets [28, 29]. These changes correlate with upregulation of key steroidogenic enzymes, including CYP17A1 and CYP11A1, and with STAR-mediated cholesterol transport, supporting increased androgen biosynthesis during follicular maturation [30] (Fig. 2). Collectively, these adaptations reflect theca interna's specialization as an active endocrine compartment.

### Origin of theca cells: insights across species

The origin of theca cells remains under active investigation, and studies in mice, cows, and humans have proposed multiple hypotheses. The complexity of theca cell differentiation and recruitment reflects their diverse roles in ovarian biology and their potential relevance as therapeutic targets in reproductive medicine. Researchers have examined the embryonic origins of theca cells using complementary approaches, including lineage tracing, cell-labeling strategies, and molecular profiling across animal species [31–33]. Genetic fate-mapping studies in mice provide the strongest evidence to date and identify contributions from mesenchymal (stromal) and neural crest-derived progenitor populations to the development of the theca layer [4]. Dye- and BrdU-based cell labeling, along with transplantation assays, has also been used to track precursor cell incorporation into developing ovarian follicles in rodent models and large animals [34, 35]. More recently, transcriptomic approaches, including bulk RNA profiling and single-cell RNA sequencing, have identified conserved gene expression signatures that distinguish theca progenitor cells from other ovarian lineages across species. In addition, immunohistochemical analyses combined with classical embryological studies have provided spatial and temporal resolution of theca cell differentiation during ovarian development in vertebrates [36–38].

### Stem cell hypothesis

Theca cells, including those described as theca stem cells (TSCs), show renewal capacity and multilineage differentiation in in vitro and in vivo studies [4]. These findings support the hypothesis that a subset of theca cells originates from ovarian stem cells. This view remains controversial. Cell isolation procedures can introduce contamination, which can lead to misidentification of TSCs or mesenchymal-like stromal cells [39]. In addition, recent studies have raised doubts about the existence and functional relevance of ovarian stem cells in adult human ovaries, suggesting that earlier reports relied on markers with limited specificity or on culture systems that introduced artefacts [7, 8].

Therefore, while the stem cell hypothesis remains a compelling research direction, the field requires rigorous validation. Future studies should identify robust, specific markers for putative TSC populations and apply lineage-tracing strategies, using improved isolation methods, to test their physiological relevance. Such work will help define the developmental origins of theca cells more conclusively.

### Embryonic progenitor cells

Another widely accepted model proposes that theca cells arise from embryonic progenitor populations. Liu et al. identified two theca cell precursors using a Gli1 + lineage-tracing model during mouse embryogenesis [40]. This work provided genetic evidence for the origin of theca cells and described multicellular interactions required to establish a functional theca layer. The authors reported that theca cells derive from ovary-derived Wt1 + cells and mesonephros-derived Gli1 + cells that migrate from the mesonephros. Paracrine signals, including Desert Hedgehog (DHH) and Indian Hedgehog (IHH), produced by GCs, induce Gli1 expression in theca progenitor cells (TPCs). Ovaries lacking Dhh/Ihh show loss or reduction of the theca layer surrounding follicles, abnormal steroid production, arrested folliculogenesis, and failure to form the corpus luteum [10, 40]. These findings indicate that factors produced by GCs at the primary and secondary follicular stages regulate recruitment of theca cells from the stroma. In the same study, oocyte-derived growth differentiation factor 9 (GDF9) regulated Dhh/Ihh production in GCs [40].

### Neural crest contribution

**Fig. 3:**
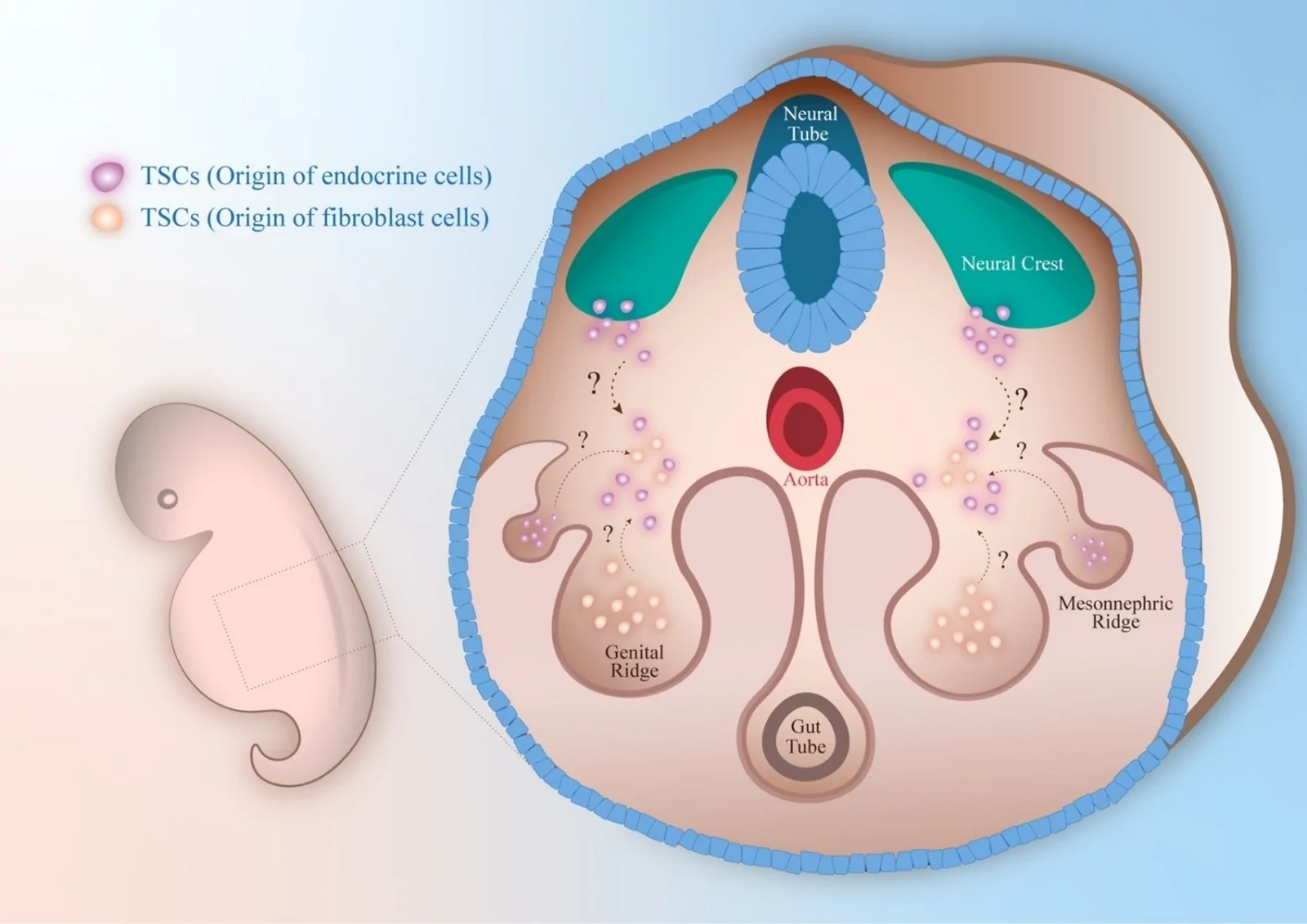
Proposed embryonic origins of TSCs in mice. The illustration shows a transverse section of an early embryo and the proposed sources of TSCs. TSCs proposed to differentiate into endocrine cells (Gli1+) (purple) are thought to originate from the mesonephric ridge, whereas TSCs proposed to give rise to fibroblast-like cells (Wt1+) (orange) are thought to arise from the genital ridge. The figure also indicates a possible contribution from neural crest cells, based on the presence of neural crest-derived cells near the anterior region of the ovary. The embryonic origin of human TSCs remains uncertain, as shown by question marks, and requires further investigation

Recent studies suggest that mouse ovaries contain TSCs [41], and some investigators have proposed a neural crest origin for at least a subset of theca cells [42]. In 2019, McKey et al. reported that neural crest cells invade the mouse ovary around embryonic day (E) 16.5. Their study described a specialized neural crest population that colonizes the ovary, differentiates into neurons and glial cells, and forms a dense neuronal network within the ovarian medulla. The authors also reported that this innervation is ovary-specific [43]. Mesenchymal-derived theca cells migrate into the mouse ovary between E17.5 and P5, beginning approximately one day after neural crest colonization [40]. These neuronal networks lie adjacent to the theca layer of growing follicles, suggesting that they may contribute to the induction of theca cells or smooth cells during follicular growth and ovulation [4, 42] (Fig. 3).

### Stromal and mesenchymal sources

Several studies indicate that bovine theca cells are recruited from surrounding stromal tissue in response to factors secreted by activated primary follicles [10, 44]. The field has not fully defined the signals that drive stromal cell differentiation into theca cells. Current models propose that interactions among granulosa cells, oocytes, and stromal cells contribute to this process. Investigators have also proposed that bovine ovaries contain stromal stem cell niches that secrete growth factors supporting the development of multiple cell types, including theca cells [1, 40, 44–46]. In related work, immunostaining for chondroitin or dermatan sulfate epitopes (antibodies 7D4, 3C5, and 4C3) identified large positively stained regions, including stromal cells surrounding large blood vessels, consistent with stem cell niches described in other tissues. These stromal cells may therefore represent a niche that gives rise to theca cells [10]. Given the importance of gene expression patterns in cell differentiation, several studies have profiled gene expression in bovine theca and granulosa cells. These profiles are highly similar, with reported correlations of 92–95%. Nevertheless, several genes, including *ACTA2, ADAM-DEC1, CLD11, LOX, OMD*, and *PDGFRα*, show differential expression in theca cells [47]. Evidence also suggests that these putative stromal niche cells express Hedgehog pathway genes, including *Ptch1, Ptch2, Gli2*, and *Gli3*, proposed as TSC lineage-associated biomarkers [10, 41]. These findings require further validation, including analyses to determine whether all or only a subset of cells within these stromal colonies express Hedgehog pathway genes. Additional studies should define the mechanisms that govern this differentiation process [42].

### Human-specific insights

In humans, Lustgarten Guahmich et al. used single-cell multiomics to map the differentiation trajectory of theca cells. They identified three theca cell subtypes, structural, androgenic, and perifollicular, and reported that these subpopulations likely arise from distinct progenitors within the ovarian stroma [48]. These findings support a model in which paracrine signals from GCs during folliculogenesis recruit theca cells from the surrounding stroma. A recent study in women also proposed that theca cells originate from the gonadal primordium or coelomic epithelium and the adjacent mesonephros, with paracrine regulation by GCs [10, 49]. Additional studies suggest that mesenchymal stem cells (MSCs) within the ovary may also give rise to theca cells. Vlieghe et al. reported that ovarian theca interna cells differentiate from MSC-like cells. This model aligns with observations across several species, including humans, in which MSCs express mesenchymal lineage markers and differentiate into TPCs in vitro. Proposed stromal niches near blood vessels may support these MSC populations, similar to stem cell niches described in other tissues [39].

The formation of the theca layer is a dynamic, species-specific process that involves multiple cellular sources, including embryonic progenitor cells, stromal cells, MSCs, and potentially neural crest cells. Future studies that integrate cytological, histological, and gene-expression analyses across developmental time points and species will help elucidate the mechanisms governing theca cell differentiation and recruitment. A clearer understanding of these processes will advance ovarian biology and support the development of targeted interventions for reproductive disorders.

### TSCs

TSCs have attracted increasing attention in reproductive biology because they may contribute to ovarian function and regenerative approaches. In 2007, Honda et al. isolated putative TSCs from neonatal mouse ovaries and reported that these cells exhibited self-renewal and differentiation capacities in vitro and in vivo [41]. These cells showed a fibroblast-like morphology, formed round colonies with a relatively homogeneous cell population, and differentiated into early progenitor and steroidogenic cells in response to serum, LH, and paracrine factors from granulosa cells. Co-culture with isolated granulosa cells promoted a mature morphology. After transplantation into the ovaries, the cells localized primarily to the ovarian interstitium and theca layer of follicles, where they differentiated. 

Subsequent studies have examined the differentiation potential of TSCs across species. Lee et al. reported that porcine ovarian theca-derived multipotent stem cells differentiated into mesenchymal lineages and oocyte-like cells, supporting their potential utility in regenerative medicine and reproductive biology research [50]. Adib et al. also identified TSCs from sheep ovarian follicles and reported differentiation into osteocyte-like, adipocyte-like, and steroid-producing cells [51]. These TSCs also expressed markers associated with germline stem cells, which suggests broader differentiation potential.

 In our previous study, hTSCs were isolated from theca layer of small antral follicles and characterized [52]. These hTSCs showed a fibroblast-like morphology and expressed MSC markers, including CD29, CD44, CD73, CD90, and CD105 [52]. The cells also showed substantial proliferative capacity, with a relatively high proportion of cells in S phase, consistent with active cycling. In addition, hTSCs differentiated into adipocyte-like, osteocyte-like, chondrocyte-like, and oocyte-like cells, providing a model for studies of germ cell formation and differentiation [52]. The functional competence of the oocyte-like cells requires further investigation. A subset of cells exhibited alkaline phosphatase activity, and their behavior resembled that of human bone marrow mesenchymal stem cells (hBMSCs). These findings aligned with observations reported by Honda et al. [41] . Recent work has also reported expression of MSC-related markers in TSCs across humans and other species, supporting a proposed MSC origin. Overall, these data indicate that TSCs share core characteristics with MSCs, including differentiation toward mesenchymal lineages, with potential relevance for tissue engineering and regenerative medicine [39].

Despite these encouraging findings, the identity and physiological relevance of TSCs, particularly in the adult human ovary, remain debated. A major limitation across studies is the risk of stromal or mesenchymal cell contamination during isolation, which can confound interpretation of differentiation assays. In addition, although in vitro plasticity and differentiation into oocyte-like or steroidogenic cells have been consistently reported, the true developmental potential and in vivo functionality of these cells remain unclear.

In addition, recent evidence has challenged the broader hypothesis that ovarian stem cells, including TSCs, contribute to adult folliculogenesis and has questioned whether such cells exist in postnatal human ovaries [7, 8]. These studies suggest that earlier conclusions relied on markers with limited specificity or on culture conditions prone to artefacts. Future work should address these limitations through in vivo lineage tracing, identification of specific and exclusive markers, and functional transplantation assays to define the existence and role of TSCs in human ovarian biology.

### Differential potential of hTSCs

#### Differentiation of theca progenitor

hTSCs show substantial differentiation capacity, particularly toward hTPCs, which contribute to ovarian function and folliculogenesis. Building on findings from Honda et al. , who reported differentiation of mouse TSCs into steroidogenic cells, subsequent studies extended these observations to human models. Dalman et al. isolated hTSCs from the theca layer of small antral follicles and induced differentiation into hTPCs using medium supplemented with LH, IGF1, and stem cell factor (SCF) [52]. During differentiation, hTPCs developed cytoplasmic lipid droplets, consistent with steroidogenic activity. Quantitative RT-PCR data showed upregulation of hTPC markers, including LHR and CYP17A1, and theca-specific effector genes GLI2 and PTCH1 [52]. These expression patterns supported differentiation of hTSCs into hTPCs.

Functionally, differentiated hTPCs secrete dehydroepiandrosterone (DHEA), a precursor of testosterone and androstenedione, and studies report increased DHEA concentrations in culture supernatants. Studies also report increased estrogen levels during hTSC differentiation into hTPCs. Detection of 3β-HSD further supported steroidogenic activity during hTSC differentiation into hTPCs.

In a recent study, the functionality of hTPCs was assessed by using them as a feeder layer to support the development of primordial follicles in human ovarian tissue [53]. Compared with control groups without a feeder layer, ovarian tissue fragments cultured with an hTPC feeder layer showed significant follicular growth and a higher number of growing follicles. Gene expression analyses showed increased expression of folliculogenesis-related genes (*ZP1, ZP2, ZP3, BMP-7, AMH*, and *GDF9*) and reduced expression of apoptosis-related genes (*P53* and *CASP3*) in the co-culture group. Hormone assays also showed higher levels of estradiol, progesterone, testosterone, and androstenedione in the co-culture group, consistent with increased steroidogenic activity. These effects likely reflect secretion of paracrine factors, including steroid and non-steroid mediators, into the culture medium. To examine this possibility, EVs were isolated from hTPCs and their effects on granulosa cells were assessed. hTPC-derived EVs improved granulosa cell responses even without direct theca cell contact, supporting a mechanism in which hTPCs promote folliculogenesis through secreted factors (unpublished data).

These findings demonstrate the capacity of hTSCs to differentiate into functional hTPCs and support ovarian follicle development. Further studies should define the specific paracrine mechanisms involved and evaluate the potential therapeutic applications of hTSCs and hTPCs in reproductive medicine.

#### Differentiation into oocyte-like cells and follicle-like structures

Recent work reported that TSCs differentiate into oocyte-like cells and follicle-like structures, with potential relevance for reproductive biology and infertility treatment [54]. In porcine models, TSCs were induced to express oocyte-associated genes, including *GDF9B, C-MOS, DAZL, VASA, ZPC, SCP3*, and *STEIIA*, consistent with differentiation toward an oocyte-like state [50]. Studies in sheep have also reported differentiation of TSCs into oocyte-like cells, supporting cross-species potential for generating germ cell-like cells [51]. In 3D culture systems containing ovarian somatic cells, hTSCs differentiated into follicle-like structures. These structures expressed germ cell markers, including DAZL and GDF9, suggesting initiation of oocyte-like differentiation.

These findings support the plasticity of TSCs and suggest potential relevance in reproductive medicine. However, the functional competence of oocyte-like cells remains unresolved, particularly their capacity to complete meiosis and support development to viable offspring. Future studies should refine differentiation protocols, define the molecular basis of TSC plasticity, and test the developmental potential of TSC-derived oocyte-like cells in vivo.

Current evidence for differentiation of TSCs into oocyte-like and follicle-like structures largely comes from in vitro studies, and in vivo developmental competence remains unproven. Many reports use limited marker expression as a proxy for functional maturity, and the absence of long-term lineage tracing or functional gametogenesis data limits interpretation. In addition, the broader concept of ovarian stem cells generating functional germ cells in adult mammals remains controversial. Recent investigations [7, 8] argue against the presence of functional germline stem cells in postnatal human ovaries and suggest that earlier reports may reflect technical artefacts or misinterpreted cellular plasticity. Therefore, while the in vitro generation of oocyte-like cells from TSCs remains a promising approach, rigorous studies should assess epigenetic stability, meiotic competence, fertilization capacity, and the ability to support embryonic development in vivo.

#### Stimulatory factors involved in theca cell recruitment and differentiation

Differentiation of theca cells from stromal precursors represents both a developmental event and a tightly regulated process controlled by paracrine and endocrine signaling. Defining the molecular cues that govern this process is essential for understanding folliculogenesis and for developing stem cell-based models of ovarian cell differentiation. Key factors that regulate theca cell function, together with representative studies across species and experimental approaches, are summarized in Table 1.

**Table 1:** Summary of key factors regulating theca cell function with representative studies across species

Factor	Primary expression site	Tested mechanisms/methods	Species	Finding	Refs.
A.IGFs	Granulosa cells (receptors present on theca cells)	*Igf1* mRNA/PCR	Mice	*IGF1* expression in theca cells promotes secondary follicle growth	[55]
		Androsterone/RIA Luteinizing hormone receptor, steroidogenesis acute regulatory protein (StAR), CYP11A, CYP17, and 3β-hydroxysteroid dehydrogenase (3β-HSD)/PCR	Rat	IGF-I is one of main regulators of TC differentiation	[56]
		Proliferation of cells/ determination of [^3^h] thymidine incorporation and cell counting	Human	IGF-I stimulates theca-interstitial cell proliferation in PCOS	[57]
		Proliferation of rat theca-interstitial (T-I) cells/DNA synthesis	Rat	IGF-I regulates DNA synthesis and steroidogenic cell proliferation via IGF receptors, modulated by IGFBP	[58]
		Cell proliferation/thymidine incorporation and cell counting	Sprague–Dawley rats	IGF-I selectively promotes proliferation of specific theca-interstitial cell subsets	[59]
		Cell numbers, steroidogenesis, and IGF-I binding sites in cultured thecal cells	Bovine	IGF-I modulates basal and LH-driven theca cell steroidogenesis and mitogenesis	[60]
B. SCF	Granulosa cells	Androsterone/RIA Luteinizing hormone receptor, steroidogenesis acute regulatory protein (StAR), CYP11A, CYP17, and 3β-hydroxysteroid dehydrogenase (3β-HSD)/PCR	Rat	SCF is important regulators of TC differentiation	[56]
		*SCF* mRNA/PCR	Human	SCF promotes theca-granulosa cell proliferation and steroidogenesis	[61]
		SCF staining	Human	SCF regulates primordial germ cell migration and early ovarian development	[62]
		Immunostaining	*Podarcis s. sicula*	*SCF* expression varies with follicular development, influencing oocyte maturation	[63]
		Cell Counting	Rat	SCF stimulates follicular growth via Erk1/2 activation in theca-interstitial cells	[64]
C. Fibroblast growth factor (FGF)	Both granulosa and theca cells	mRNA Detection/PCR	*cattle*	*FGFR1c/FGFR4* upregulation in theca cells suggests FGF’s role in dominant follicle selection	[65]
		Immunohistochemistry	porcine	*FGF7* is expressed in theca cells during in vitro maturation	[66]
D. TGFβ	Theca cells and stromal cells	mRNA Detection/PCR	Human	GDF9 activates ALK5-SMAD2/3 pathway, suppressing CYP17A1 and androgen production in human theca cells	[67]
E. Inhibin	Granulosa cells	mRNA Detection/PCR	Cattle	Inhibins indirectly enhance theca cell androgen production by antagonizing BMPs	[68]
		Northern blot	Human	Inhibin isoforms are present in follicular fluid	[69]
F. Activin	Granulosa cells	Northern blot	Human	Activin isoforms are present in follicular fluid	[69]
G. BMPs	Both granulosa and theca cells (species and type dependent)	mRNA Detection/PCR	Human	hTPCs regulate primordial follicle growth via *ZP, BMP-7, AMH,* and *GDF9* expression	[53]
		Cell proliferation, androgen production, expression of androgen-synthesizing genes, inflammatory gene expression/Cell counting & PCR	PCOS Cell Model	BMP-2 regulates theca steroidogenesis and is a potential PCOS treatment target	[70]
H. GDF-9	Oocytes	Immunoblot	Immature rats	Increase of CYP17, a theca cell marker, in the ovarian homogenate after treatment with GDF-9	[71]
		Immunofluorescence	Immature KM mice	GDF-9 dose-dependently enhances mouse theca interstitial cell number and viability	[72]
I. HH	Granulosa cells (receptors expressed on theca cells)	mRNA Detection/PCR	Mouse	Hedgehog signaling induces *Ptch1* and *Gli1* expression in pre-theca cells	[73]
		Semi-quantitative RT-PCR and northern blot analyses	Mice, rat, and bovine	HH from granulosa cells activates Ptch1 and Gli1 in theca cells, promoting proliferation and androgen synthesis in bovine follicles	[74]
		Cell-fate mapping	Mice	HH signaling promotes vasculature development and steroidogenesis in theca during follicle growth	[75]
		mRNA Detection/PCR	Mouse	Hedgehog target genes *Ptch1* and *Gli1* are highly expressed in pre-theca cell compartment	[73]

#### Insulin-like growth factors (IGFs)

IGFs, particularly IGF1, regulate ovarian folliculogenesis and play key roles in controlling the proliferation and steroidogenic activity of theca cells. The liver produces IGF1 in response to growth hormone (GH), and IGF1 acts systemically through endocrine signaling. Within the ovary, IGF1 promotes theca cell proliferation and stimulates steroidogenesis. In vitro studies report that FSH-regulated IGF1 production by GCs promotes theca cell proliferation [76–78]. In addition, IGF1 acts synergistically with luteinizing hormone (LH) to increase androgen production in theca-interstitial cells through activation of IGF1 receptor (IGF1R)-mediated signaling, particularly the PI3K/Akt pathway, which supports cell growth and differentiation [79]. IGF1 also promotes intercellular communication by increasing gap junction formation between follicular cells. GH indirectly modulates theca cell function by inducing hepatic IGF1 production, although direct ovarian effects of GH remain unclear [80]. Evidence suggests that GH increases follicular sensitivity to gonadotropins, thereby strengthening LH- and FSH-dependent steroidogenesis and follicular development [79](Table 1A).

#### SCF

Stem cell factor (SCF), also known as Kit ligand, is produced mainly by GCs and signals through the c-Kit receptor expressed on theca cells, stromal cells, and oocytes [81]. The SCF/c-Kit signaling axis regulates recruitment, proliferation, and differentiation of theca cells during follicular development [15, 82, 83].

Functional studies report that neutralization of SCF and IGF1 in follicle-conditioned media markedly reduces stimulation of theca cell differentiation, supporting a central role for SCF in this process [1]. In rat theca cells, SCF and IGF1 increase androgen production, although their effects are weaker than those of LH. SCF alone reduces CYP17 expression without major changes in other steroidogenic genes, whereas IGF1 increases expression of several key steroidogenic regulators. When combined, SCF and IGF1 act synergistically by increasing the expression of genes involved in theca cell function. In bovine cell cultures, SCF stimulates the growth of theca and stromal cells under subconfluent conditions and increases androstenedione production in confluent monolayers [84]. In bovine stromal cells, SCF does not alter hCG-induced androgen secretion, which suggests species-specific differences in pathway interactions [85]. LIF, KGF, and HGF regulate SCF expression, forming a network that supports theca cell differentiation [4, 86] (Table 1B).

#### Fibroblast growth factor (FGF)

FGFs, including basic FGF (bFGF/FGF-2) and FGF-9, regulate granulosa and theca cell proliferation and differentiation. bFGF is expressed in oocytes, granulosa cells, and theca cells, and its receptors mediate paracrine signaling. bFGF mRNA levels decrease as follicles mature, which suggests a primary role during early folliculogenesis [87]. In addition, GC-derived Kit ligand induces fibroblast growth factor-7 (FGF-7) production, and localization studies suggest that FGF-7 contributes to early recruitment and development of theca layer by acting on theca precursor cells [88]. GC-secreted FGF-2 may also regulate angiogenesis and support incorporation of newly formed capillaries into the theca layer [89]. FGF-9, expressed mainly in theca and stromal cells, promotes proliferation of bovine theca cells while inhibiting differentiation by reducing expression of key steroidogenic genes, including steroidogenic acute regulatory protein (StAR) and insulin-like growth factor 2 (IGF-2) [90]. This dual effect positions FGF-9 as a negative regulator of differentiation, supporting cell growth while limiting steroidogenesis.

FGF-9 also regulates granulosa cell function. Oocytes produce FGF-9 in primordial and primary follicles, and granulosa cells in larger preantral follicles and theca layer also express FGF-9 across follicle stages. Expression of FGF receptors in both granulosa and theca cells supports these interactions. In cultured rat granulosa cells, FGF-9 increases progesterone synthesis by increasing expression of steroidogenic enzymes, including StAR and P450 side-chain cleavage enzyme (P450scc) [91, 92]. 

In addition, culture studies indicate that FGF-9 promotes primordial follicle activation and supports the transition to primary follicles [91]. However, bFGF-null mouse models suggest that FGF signaling supports folliculogenesis but is not essential for this process [93]. Overall, members of the FGF family exhibit stage-specific effects that balance the proliferation and differentiation of theca cells (Table 1C).

#### Members of the TGF-β superfamily

The TGF-β superfamily comprises a diverse group of proteins, including bone morphogenetic proteins (BMPs), growth differentiation factors (GDFs), anti-Müllerian hormone (AMH), activins, and inhibins, which regulate theca cell function and androgen synthesis. These factors support granulosa cell proliferation, promote follicular survival, and limit premature luteinization or atresia. Oocytes secrete TGF-β family members, notably BMP15 and GDF9, which stimulate cyclin D2 expression and DNA synthesis in granulosa cells. GDF9 is essential for recruitment, proliferation, and functional development of theca cells and increases theca-interstitial cell proliferation and androgen production [94–96]. Activins and inhibins also regulate steroidogenesis. Activins suppress androgen synthesis by reducing the expression of steroidogenic enzymes, whereas inhibins antagonize activin signaling and thereby promote androgen production [97, 98] (Table 1D).

##### Inhibin and activin

The intra-ovarian roles of activin and inhibin extend beyond granulosa cells and are increasingly recognised as key regulators of the stromal and theca compartments [99]. Activin, produced mainly by granulosa cells, exerts pleiotropic effects within the ovary, including regulation of primordial follicle assembly, germ cell maintenance, and follicular growth in humans [100] and animal models [101].

Within theca compartment, activin directly suppresses steroidogenesis by reducing expression of key steroidogenic enzymes, including cytochrome P450 17A1 (CYP17A1) and 3β-hydroxysteroid dehydrogenase (HSD3B) [102, 103]. This suppression reduces androgen and progesterone synthesis and lowers androstenedione secretion [97]. Alongside this inhibitory effect on steroid production, activin promotes proliferation of thecal-interstitial and stromal cell populations, supporting a role in recruiting and expanding the theca lineage during follicular development [104]. 

As follicles approach the preovulatory stage, rising estradiol and inhibin levels progressively reduce activin bioavailability, establishing an intrafollicular feedback loop [97]. Inhibin antagonizes activin signaling through betaglycan-dependent receptor sequestration [105, 106], which alleviates activin-mediated suppression of steroidogenic enzyme expression in theca cells [97–107]. This shift increases androgen production and supports the steroidogenic changes required during late follicular maturation [108].

Collectively, these findings indicate that the activin–inhibin system regulates the recruitment and proliferation of theca or stromal precursors and the steroidogenic differentiation of mature theca cells. These data support activin–inhibin signaling as a direct regulator of theca cell lineage specification and functional maturation, rather than a pathway acting exclusively through granulosa cells (Table 1E, F).

##### BMPs

BMPs are produced at specific stages of folliculogenesis and regulate growth, differentiation, and function of follicular cells through autocrine and paracrine mechanisms. BMP expression profiles vary across species, including ruminants, rodents, and primates, and several BMPs show species-specific patterns [109, 110].

BMP-4 is expressed in stromal cells surrounding primordial follicles, whereas BMP-7 localizes to the theca layer of antral follicles [1, 111]. In vitro, BMP-4 reduces androstenedione production in human theca-derived tumour cells, and cAMP agonists strengthen this effect [112]. In addition to effects on steroidogenesis, BMP-4 supports follicle survival and promotes the transition from primordial to primary follicles, which supports early follicle development [113]. ​

Studies in ovine and bovine theca cells treated with BMP-2, BMP-4, BMP-6, and BMP-7 reported reduced androstenedione secretion and CYP17 expression, along with a modest increase in progesterone production and cell proliferation [114, 115]. BMP-6 suppresses both basal and LH-induced androgen production, which supports a role in modulating steroidogenesis within the ovarian follicle.

BMP-15, primarily expressed in oocytes, is required for follicle growth and maturation. It regulates granulosa cell sensitivity to FSH and influences ovulation rate. Mutations or dysregulation of BMP-15 and related signaling components have been associated with reproductive disorders, including premature ovarian failure and PCOS [116–118]. Collectively, BMPs regulate recruitment, specification, and functional differentiation of theca cells, which highlights their relevance for approaches aimed at restoring or engineering functional theca layers (Table 1G). 

##### GDF-9 

GDF9, produced mainly by oocytes, is required for primary follicular growth and recruitment of theca cells. Loss-of-function mutations in GDF9 cause follicular arrest at the primary stage in mice, sheep, and humans and have been associated with premature ovarian failure [1, 119–121]. In vitro, GDF9 stimulates the proliferation of bovine theca cells from small follicles while suppressing steroidogenic differentiation through reduced expression of CYP17A1 and 3β-HSD [122]. In addition, GDF9 reduces LHR expression and LH-induced steroidogenesis without changing IGF1R or StAR levels, which suggests that it maintains theca cells in a proliferative, less differentiated state [122, 123].

GDF9 acts synergistically with BMP-15 to regulate granulosa cell proliferation and, indirectly, theca cell development, in part by modulating SCF expression [1, 124, 125]. Overall, GDF9 functions as a central oocyte-derived regulator of theca cell recruitment and maturation and represents a key factor for reconstructing theca–granulosa–oocyte interactions in vitro (Table 1H).

#### Hedgehog (HH) proteins

Hedgehog (HH) proteins, particularly Desert Hedgehog (DHH) and Indian Hedgehog (IHH), regulate ovarian folliculogenesis and play important roles in the recruitment and differentiation of theca cells.

In the murine ovary, GCs begin expressing HH ligands at the early primary follicle stage in response to oocyte-derived GDF9. These ligands bind Patched receptors (PTCH1 and PTCH2) on adjacent mesenchymal cells, relieve inhibition of Smoothened (SMO), and activate downstream signaling through GLI transcription factors. This signaling promotes expression of HH target genes, including Gli1, Ptch1, and Hhip, which support theca cell differentiation [73, 75].

Activation of HH signaling by DHH and IHH promotes recruitment of mesenchymal precursors to the follicle and supports their differentiation into functional theca cells, as shown by increased expression of HH-responsive genes (Ptch1, Ptch2, Hip1, Gli1) in developing theca cells [75].

HH signaling also interacts with other pathways, including FGF receptor 2, BMPs, and additional regulatory networks [126, 127], forming an interconnected signaling landscape that shapes follicular development [73]. Although oocytes do not express HH receptors, pretheca cells express HH target genes, which function as markers of this compartment. Mouse models with constitutive HH activation exhibit defective theca development, including reduced or absent smooth muscle, indicating that folliculogenesis requires tightly controlled HH activity [1, 128].

HH signaling primarily promotes commitment of stromal progenitors to theca lineage, whereas TGF-β and BMP pathways modulate expression of steroidogenic genes (CYP17A1, CYP11A1, HSD3B) [1, 15, 73–75, 129, 130]. In parallel, IGF–PI3K–AKT signaling increases androgen synthesis and modulates cellular metabolism linked to LH responsiveness. Crosstalk among these pathways coordinates recruitment (HH), differentiation (TGF-β/BMP), and metabolic support (IGF), sustaining steroidogenic output and supporting folliculogenesis (Table 1I).

#### Theca cell function

Theca cells are integral components of ovarian follicles and contribute to follicle growth, steroid hormone production, and female reproductive function. Their roles change throughout folliculogenesis, reflecting functional specialization across stages.

During early follicle development, theca cells are recruited from surrounding stromal tissue and acquire LH receptors, which enable androgen synthesis, primarily androstenedione, which granulosa cells subsequently convert to estrogens [131–133]. In humans, androgen production follows the Δ5 steroidogenic pathway and supports local follicular function and systemic reproductive processes [10, 134].

As follicles reach the antral stage, theca cells sustain LH-stimulated androgen synthesis and provide structural support to the follicle, which promotes antral cavity formation and supports nutrient and hormone exchange [135, 136]. After ovulation, theca cells luteinize into theca lutein cells within the corpus luteum and produce progesterone and androgens that support endometrial preparation and early pregnancy [137].

In addition to steroidogenesis, theca cells contribute to oocyte maturation by supporting cyclic nucleotide signaling (cAMP, cGMP) through gap junction-mediated communication, which helps maintain meiotic arrest until ovulatory cues occur [138]. Theca cells also express luteotropic factors, including LPA and its related enzymes and receptors, and AMH receptor type II (AMHRII), which enables them to respond to intra-ovarian signals that regulate androgen production and follicular progression [139–142]. Species differences exist. For example, porcine theca cells express AMH, which suggests variation in regulatory mechanisms across mammals [143].

#### Paracrine interactions between theca cells, granulosa cells, and oocytes in follicular development

Theca cells interact dynamically with granulosa cells and oocytes to support follicle growth, maturation, and steroidogenesis. In mice, theca cells become morphologically distinguishable during the transition from primary to secondary follicles, coinciding with the expansion of granulosa cell layers [40]. In humans, theca cells first appear at the secondary follicle stage, underscoring their coordinated emergence with follicular maturation [15].

Paracrine communication increases steroidogenic capacity in theca cells [131, 144]. Co-culture studies show that granulosa cell-derived signals increase expression of steroidogenic enzymes and enhance LH receptor responsiveness in theca cells, which promotes androgen synthesis [144, 145].

Similarly, interactions with oocyte–cumulus–granulosa complexes (OCGCs) support theca-mediated androgen production, which is required for oocyte growth and subsequent embryonic developmental competence [146].

Beyond steroidogenesis, theca cells contribute to extracellular matrix remodelling and angiogenesis, in part through secretion of TGF-β and other modulatory factors [147]. Their gene expression profiles, including collagen- and integrin-associated genes, reflect structural and functional specialization within the follicle [148]. These findings highlight the importance of intercellular signaling in coordinating follicle development and supporting proper oocyte maturation.

#### Vascularization in the theca layer

**Fig. 4:**
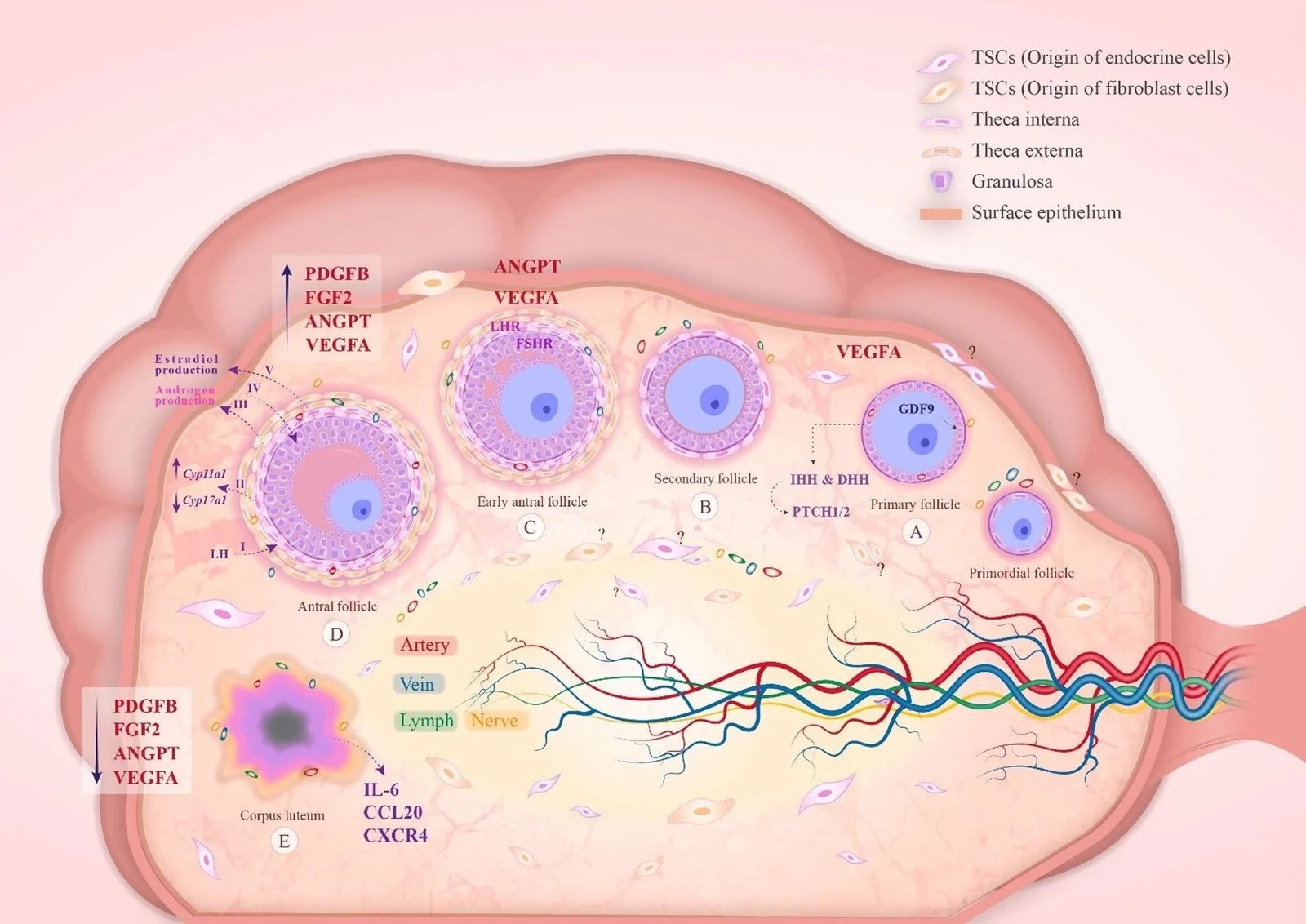
Vascularization dynamics in the theca layerduring follicular development and luteinization. The illustration depicts vascular developmentthat supports ovarian follicles across stages of maturation. During early stages, primordial andprimary follicles (A) rely mainly on stromal blood vessels for nutrient and oxygen supply. Asfollicles reach the secondary stage (B), angiogenic factors such as vascular endothelial growthfactor A (VEGFA), fibroblast growth factor 2 (FGF2), and angiopoietins (ANGPTs) are secretedand stimulate angiogenesis in the surrounding theca layer. This vascular expansion supportsdelivery of hormones, oxygen, and metabolic substrates to growing follicles. With transition tothe early antral (C) and antral (D) stages, angiogenesis intensifies further, supports antrumformation, and meets the metabolic demands of follicular maturation and ovulation. Growthfactors such as platelet-derived growth factor subunit B (PDGFB), FGF2, ANGPTs, and VEGFAcoordinate vascular remodelling during these stages. After ovulation, formation of the corpusluteum (E) involves rapid angiogenesis, which supports luteal function. Inflammatory mediatorssuch as interleukin-6 (IL-6), C-C motif chemokine ligand 20 (CCL20), and C-X-C chemokinereceptor type 4 (CXCR4) further regulate vascularization and support progesterone secretionrequired for early pregnancy. The diagram also highlights organisation of arteries, veins,lymphatic vessels, and nerves within the ovarian stroma. Question marks indicate areas wheremechanisms of vascular regulation remain incompletely defined

Angiogenesis within the theca layer supports follicular development, steroidogenesis, and ovulation, and it is required for corpus luteum formation. This vascular network delivers nutrients, oxygen, gonadotropins, and cholesterol substrates to metabolically active follicular cells, supporting reproductive function (Fig. 4).

During the primordial and primary stages, follicles remain avascular and rely on stromal blood vessels for metabolic support [149, 150]. As follicles enter the secondary stage, theca layer begins to form and releases paracrine signals that act on stromal endothelial cells. GCs and oocytes increase secretion of angiogenic factors, particularly VEGF and bFGF, which initiates vascular ingrowth into the theca layer [150]. This process disrupts the basement membrane of adjacent vessels and allows endothelial cells to migrate into the developing theca interna.

At the antral stage, angiogenesis intensifies and forms a dense capillary network within the theca interna. This vascularization supports delivery of hormonal signals (e.g., LH), low-density lipoproteins, and oxygen, which are required for steroid biosynthesis and follicular growth. VEGF plays a central role at this stage by stimulating endothelial proliferation and increasing vascular permeability, which supports follicular fluid accumulation and antrum formation [151, 152].

Postovulation, the residual follicle rapidly remodels into the corpus luteum, a process associated with one of the highest angiogenic rates in adult tissues. Theca-derived endothelial and pericyte progenitors contribute to the vascular framework of the corpus luteum. The extent of vascularization influences luteal progesterone output and, in turn, supports early pregnancy [152]. Disruption of theca layer vascularization during this phase can contribute to luteal insufficiency or early pregnancy loss.

Angiogenesis is tightly regulated by interactions between proangiogenic factors (e.g., VEGF, bFGF, angiopoietins) and antiangiogenic factors (e.g., thrombospondins, angiostatin), and theca cells both respond to and contribute to this local angiogenic environment. Gonadotropins, particularly LH, induce VEGF expression in GCs and possibly in theca cells, creating a hormonal–vascular feedback loop.

Emerging studies suggest that dysregulated theca layer angiogenesis contributes to disorders such as PCOS, in which abnormal vascularization associates with follicular arrest and hyperandrogenism [154, 155]. In contrast, improved angiogenic responses may support outcomes in ART. Defining mechanisms of theca angiogenesis, therefore, links basic ovarian biology with clinical strategies in infertility treatment and contraception.

#### Theca cell dysregulation in ovarian pathologies

Theca cells support normal ovarian function by regulating follicular development and steroid hormone synthesis. Increasing evidence links theca cell dysregulation to several ovarian pathologies. Abnormal theca cell activity, including excess androgen production, impaired steroidogenesis, and altered paracrine signaling, has been implicated in PCOS, ovarian hyperthecosis, and POI. Defining how intrinsic and extrinsic factors alter theca cell program provides insight into disease mechanisms and positions theca cells as potential therapeutic targets.

Beyond nutrient and hormone delivery, evidence indicates that endothelial–theca cell crosstalk actively regulates steroidogenesis and extracellular matrix remodelling. VEGF signaling through VEGFR2 on endothelial cells drives vascular expansion and can influence adjacent theca cells by increasing oxygen availability and cholesterol transport, thereby supporting androgen biosynthesis [155]. In parallel, angiopoietin–TIE2 signaling contributes to vascular stabilization and pericyte recruitment, which helps maintain structural integrity of the theca layer during follicular growth and luteinization. 

Immune cells, particularly ovarian macrophages within the theca and stromal compartments, further integrate vascular and endocrine signaling. Macrophage-derived cytokines such as IL-10 support tissue remodelling and angiogenic stability, whereas TNF-α regulates matrix metalloproteinase (MMP) activity and steroidogenic enzyme expression in theca cells [156, 157]. Through these paracrine interactions, endothelial and immune cells collectively influence the mechanical properties, vascular permeability, and steroidogenic capacity of the theca layer.

Thus, angiogenesis within the theca should be regarded not only as a supportive process but as an integral component of a multicellular regulatory network that links vasculature, immune cells, and steroidogenic theca cells to coordinate follicular growth, ovulation, and luteal function.

#### PCOS

Studies using primary theca cell cultures consistently indicate that intrinsic theca cell dysfunction contributes to the hyperandrogenism observed in women with PCOS. The landmark study by Gilling-Smith et al. (1994) reported that theca cells from polycystic ovaries secreted significantly higher levels of androstenedione than those from normal ovaries, under both basal conditions and after LH stimulation [11]. This increased androgen output persisted in vitro, which indicates that the steroidogenic abnormality does not depend on in vivo endocrine or metabolic influences and instead reflects an intrinsic defect in theca cells. The elevated androstenedione-to-progesterone ratio in polycystic ovary-derived theca cells also suggests an altered steroidogenic enzyme profile that favors androgen biosynthesis.

Subsequent studies using cultured human theca cells and ovarian tissue have reinforced these conclusions by showing dysregulated androgen metabolism and increased androgen production in PCOS ovaries. Increased 5α-reductase activity within follicles from women with PCOS increases intragonadal conversion of testosterone to the more potent dihydrotestosterone (DHT), which can amplify local androgen signaling even without marked rises in circulating androgens [158]. More recent multi-omics and functional studies indicate that intrinsic theca cell alterations extend beyond changes in steroidogenic enzyme expression and include dysregulation of broader metabolic and signaling pathways linked to follicular function and ovarian tissue remodelling [159–161]. These datasets suggest that theca cell dysfunction in PCOS drives excess androgen output and alters mechanotransduction, ECM remodelling, and stromal–immune interactions, processes increasingly associated with follicular arrest and changes in ovarian microarchitecture in PCOS.

PCOS also involves altered ovarian responsiveness to gonadotropins and metabolic dysregulation, most notably hyperinsulinemia. Insulin stimulates androgen production in both normal and polycystic ovaries and, together with LH, strongly increases steroidogenesis [162]. Although PCOS is associated with systemic insulin resistance, theca cells retain insulin signaling and often show heightened responsiveness. Studies using human theca cell culture models report exaggerated androgen production in response to combined LH and insulin stimulation, mediated in part through altered PI3K signaling [163]. Together, these findings indicate that PCOS reflects the convergence of endocrine (LH), metabolic (insulin), and intrinsic abnormalities of the theca cells.

PCOS is a common gynaecologic disorder that affects up to 13% of women of reproductive age and represents a leading cause of infertility [164]. Clinically, PCOS is defined by a triad of ovulatory dysfunction, hyperandrogenism, and polycystic ovarian morphology [165, 166]. Although the aetiology remains unclear, genetic and environmental factors contribute to disease development.

Theca cell dysfunction contributes directly to PCOS pathogenesis. Increased production of androgens, including dehydroepiandrosterone and 17-OH progesterone, by ovarian theca cells represents a hallmark of the disorder [5, 158]. Several intrinsic and extrinsic factors, including hyperinsulinemia, elevated LH with relatively low FSH, chronic low-grade inflammation, and altered intra-ovarian paracrine signaling, promote theca cell hyperstimulation and androgen excess [5, 167]. Studies have shown that theca cells isolated from follicles of women with PCOS produce higher androgen levels than theca cells from women without PCOS undergoing in vitro fertilization (IVF). This increased androgen production persists after multiple in vitro passages [168], supporting the concept that intrinsic changes in theca cell steroidogenesis persist independently of systemic influences.

Compared with theca cells from women without PCOS, theca cells from women with PCOS produce higher levels of androgens. At the molecular level, PCOS theca cells show altered expression of key steroidogenic enzymes, including CYP17A1, CYP11A1, and HSD3B2, which increases androgen biosynthesis [169]. Insulin also acts synergistically with LH to further increase androgen production in PCOS theca cells, which links metabolic dysregulation to ovarian steroidogenesis [15, 162, 166, 170]. Transcriptomic analyses show abnormal expression of genes involved in cell signaling, inflammation, and steroidogenesis in PCOS theca cells, including WNT5A, VCAM1, TM4S3, and DENND1A.V2, a PCOS susceptibility gene variant [15, 171].

Autophagy dysregulation has also been implicated in theca cell pathology in PCOS. Immunohistochemical analyses reported increased ubiquitin and p62 levels in theca cells from women with PCOS. In bovine theca cells, chloroquine and palmitic acid inhibited autophagy and increased ubiquitin and p62 levels, together with increased expression of CYP17A1 and plasminogen activator inhibitor-1 (PAI-1). These changes coincided with ROS generation and activation of p38 and JNK signaling. Inhibition of p38 and JNK reduced PAI-1 and CYP17A1 expression [172]. Together, these findings suggest that impaired autophagy contributes to PCOS pathogenesis through ROS- and JNK or p38-mediated induction of PAI-1 and CYP17A1.

Emerging evidence also suggests a role for dysregulated angiogenesis in PCOS, in which abnormal vascularization of the theca layer may worsen follicular arrest and anovulation [173, 174].

Genetic factors also contribute, and variants in genes involved in steroidogenesis and insulin signaling have been implicated in PCOS pathogenesis [163]. Among steroidogenesis-related genes, polymorphisms in CYP11A1, CYP17A1, and CYP19A1 have been associated with increased androgen levels and hormonal imbalance [175–180]. Within insulin signaling pathways, polymorphisms in INSR, IRS1, IRS2, and CAPN10 have been linked to insulin resistance and increased susceptibility to PCOS [182–188].

These genetic variants can disrupt steroid hormone synthesis and insulin signaling and contribute to PCOS development and progression. PCOS is a multifactorial disorder in which metabolic, endocrine, and paracrine dysregulation converge on intrinsic theca cell hyperfunction. Further studies of intrinsic abnormalities in PCOS theca cells should help identify therapeutic targets to restore normal ovarian function.

#### Hyperthecosis

Hyperthecosis is distinct from PCOS and is characterized by luteinization of the ovarian stroma, in which stromal cells differentiate and form nests or islands of hyperplastic, luteinizing theca cells distributed throughout the ovary, rather than being confined to the perifollicular region [189, 190]. Histologically, these nests comprise polygonal luteinized theca cells with eosinophilic or clear cytoplasm and are typically < 1 cm in diameter. These cells produce high androgen levels, with serum concentrations often exceeding 150 ng/dL, higher than levels commonly reported in PCOS [191]. Elevated testosterone can lead to clinical signs of virilization.

Excess androgen production appears to involve the differentiation of ovarian interstitial cells into steroidogenic, luteinized stromal cells [192]. LH stimulates stromal proliferation and androgen synthesis, and insulin resistance may contribute because insulin stimulates ovarian stromal androgen production [193]. Some evidence also suggests a genetic predisposition to hyperthecosis.

Compared with PCOS, the higher androgen levels in hyperthecosis often cause more pronounced virilizing features, including hirsutism, clitoromegaly, voice deepening, and male-pattern baldness [194]. Moreover, peripheral aromatization of excess androgens increases estrogen levels, which can lead to hyperestrogenism and raise the risk of estrogen-dependent endometrial disorders, including endometrial polyps, endometrial hyperplasia, and endometrioid adenocarcinoma, particularly in postmenopausal women [195]. Studies report a higher frequency of ovarian hyperthecosis in postmenopausal women with endometrial polyps, hyperplasia, and endometrioid adenocarcinoma than in those with atrophic endometrium [195, 196].

Given the risk of severe hyperandrogenism, virilization, and increased rates of endometrial cancer and metabolic complications, including insulin resistance and type 2 diabetes [197], accurate diagnosis and careful clinical management remain essential in hyperthecosis. Diagnosis often requires histological evaluation of ovarian tissue. Medical management with antiandrogen therapy can reduce hyperandrogenic symptoms, whereas definitive treatment, particularly in postmenopausal women, often involves bilateral oophorectomy [198].

#### POI

Primary ovarian insufficiency (POI) refers to the loss of ovarian function before age 40 and is associated with infertility, menstrual disturbance (amenorrhea or oligomenorrhea for more than four months), elevated FSH and LH levels, and low estradiol levels [199]. Menopause typically occurs between the ages of 49 and 51 [15], whereas POI leads to premature ovarian failure and has major consequences for reproductive health.

The aetiology of POI is heterogeneous and includes autoimmune disorders, environmental factors, and genetic mutations. Mutations in genes encoding pituitary hormones (e.g., FSHB) and ovarian receptors (e.g., LHCGR, ESR1) contribute to disease risk [200, 201]. For example, inactivating mutations in FSHB have been associated with primary amenorrhea, absent breast development, low FSH, undetectable estradiol, and elevated LH [15, 202]. Defects in estrogen receptor alpha (ESR1), including those affecting theca cells, also impair follicular development and fertility [203]. In Lhcgr knockout mouse models, loss of LH signaling disrupts follicular growth and leads to premature ovarian failure, underscoring the importance of LH receptor activity, particularly in theca cells [203].

Emerging evidence also implicates theca cell dysfunction in POI. Reduced insulin-like peptide 3 (INSL3), produced mainly by theca cells, has been reported in patients with POI, which suggests impaired theca cell function [6]. Because INSL3 supports follicular survival and maintenance of ovarian function, reduced INSL3 may contribute to follicular atresia and ovarian insufficiency.

FSH and LH also coordinate theca cell steroidogenesis. FSH promotes inhibin production and supports LH receptor expression in theca cells, which increases LH-stimulated androgen synthesis; disruption of this pathway can severely impair follicular development [15, 204].

Current management of POI relies mainly on hormone replacement therapy to restore endocrine balance and alleviate symptoms, but these approaches show limited efficacy in restoring fertility. As a result, regenerative strategies, including stem cell–based therapies, are under investigation as potential alternatives.

Earlier studies using bone marrow-derived stem cells (BM-MSCs) or umbilical cord-derived stem cells reported mixed outcomes and often did not restore fertility in the long term. More recent work has focused on ovarian tissue-derived stem cells, including TSCs, as potential therapies for POI. In mouse and nonhuman primate models of POI, transplantation of TSCs was reported to generate functional theca cells, support folliculogenesis, and improve fertility outcomes [205]. In these studies, TSC-derived cells contributed to androgen production and supported granulosa cell function, both of which are required for follicle maturation.

Nevertheless, challenges remain. Although studies report improved ovarian function and follicular development after TSC transplantation, these effects appear transient, with ovarian support lasting only a few months. Concerns also remain regarding chromosomal integrity of oocytes from treated ovaries and the capacity to achieve full-term pregnancies. Future studies should refine transplantation protocols, extend the durability and function of regenerated follicles, and establish safety and efficacy before translation to clinical use in humans.

To provide a concise overview of these pathological changes, Table 2 summarizes key molecular markers of theca cell function and their associations with specific disorders.

**Table 2:** Key theca cell markers and their roles in function and associated ovarian pathologies

Marker	Role in theca cell function	Associated pathologies	Refs.
CYP17A1	Enzyme catalyzing 17α-hydroxylase and 17,20-lyase activities essential for androgen biosynthesis	PCOS, hyperandrogenism	[206–208]
LHCGR (LH receptor)	Mediates LH signaling critical for theca cell steroidogenesis and follicular maturation	PCOS	[209]
INSL3	Involved in theca cell differentiation and follicle maturation	Premature ovarian insufficiency (POI), ovarian dysgenesis	[210–212]
GDF9	Oocyte-derived paracrine factor regulating theca cell function and folliculogenesis	PCOS, follicular arrest	[213, 214]
AMH (Anti-Müllerian Hormone)	Modulates follicle recruitment and influences theca cell steroidogenesis	PCOS	[215]
HSD3B2	Enzyme involved in progesterone synthesis, contributing to steroid hormone production in theca cells	PCOS, hyperandrogenism	[206, 216, 217]
IGF1R	Receptor mediating insulin-like growth factor 1 signals that enhance steroidogenesis and theca cell proliferation	PCOS, ovarian dysfunction	[218, 219]
AR (Androgen Receptor)	Mediates androgen signaling in theca cells affecting steroidogenic enzyme expression	PCOS, androgen excess	[220–222]
VEGFA	Regulates angiogenesis in theca layer supporting follicular growth and steroidogenesis	PCOS	[223, 224]
TGF-β	Modulates extracellular matrix remodeling and theca cell proliferation	Ovarian fibrosis, PCOS	[95, 225–227]

#### Theca cells and ovarian aging

Interest in how aging affects theca cell morphology and function has increased [228–230]. Ovarian aging disrupts coordination among follicular cell types, including theca cells and developing follicles. Studies report age-associated histological reorganisation of theca interna, theca externa, and surrounding interstitial tissue, which alters the microenvironment of developing follicles [231, 232]. This structural remodelling often coincides with reduced CYP11A1 expression in antral follicles of aged ovaries, as reported in chinchilla rabbits [15, 233]. More recent work also suggests that aging alters theca cell function and reduces ovarian vascularity, which can limit nutrient and hormone exchange and contribute to ovarian dysfunction [5]. These changes can impair fertility through reduced oocyte quality, disrupted folliculogenesis, and increased risk of age-related infertility.

Age-related changes in theca cell function can have substantial downstream effects. Reduced theca cell function can limit androgen availability, which granulosa cells convert into estrogens required for oocyte maturation; this reduction can contribute to poorer oocyte quality and lower developmental potential. Theca cells also support follicular structure, and impaired theca function can compromise follicle integrity, disrupt follicle growth, and interfere with ovulation. In parallel, reduced androgen production can contribute to hormonal imbalance, which can present as irregular cycles, anovulation, and reduced fertility. Together, reduced oocyte quality, disrupted folliculogenesis, and hormonal imbalance increase the risk of age-related infertility. Outcomes after assisted reproductive technologies, including in vitro fertilization, can also decline with reduced oocyte quality and impaired follicular support. Beyond fertility, endocrine changes during ovarian aging can influence broader aspects of women’s health and associate with increased risk of age-related conditions.

A key factor implicated in ovarian aging is VEGF within the follicular environment. Studies suggest that theca cells and GCs increase VEGF expression as a compensatory response to hypoxia associated with reduced vascularity in the aging ovary [78, 234]. This response may not restore sufficient oxygenation, which can promote oxidative stress and cellular damage, including mitochondrial structural changes, particularly in GCs [78, 235]. Hypoxia-associated theca cell dysfunction may also contribute to age-related remodelling of the theca layer.

Unpublished data from a study assessing theca progenitor markers (Gli1 and Wt1) and functional genes (3BHsd and Lhr) in ovaries from young (2-month), middle-aged (7-month), and old (14-month) mice showed a significant age-related reduction in the number of theca cell layers. Gli1 and Wt1 expression was lower in the 14-month group than in the 7- and 2-month groups. Expression of key steroidogenic genes, including 3BHsd and Lhr, also declined progressively with age, consistent with reduced theca cell function and impaired oocyte quality.

In a separate study, TSCs isolated from a 38-year-old woman retained the capacity to differentiate into germ cell-like cells and follicle-like structures, comparable to TSCs derived from younger ovaries (19- and 20-year-old donors) [54]. These findings suggest that aging reduces theca progenitor-associated gene expression and steroidogenic capacity, while some regenerative potential persists at later reproductive ages.

Another feature of ovarian aging is reduced androgen production by theca cells. In fertile women, preantral theca cells provide androgen support to developing follicles in response to inhibin B [236, 237]. As the follicular reserve declines with age, inhibin B levels fall, which is associated with reduced DHEA production [238]. DHEA, produced by theca cells within the ovary, contributes to fatty acid metabolism and lipid transport [239]. DHEA also supports oocyte cytoplasmic integrity, which is important for follicle survival [19]. Reduced DHEA availability can reduce activation of peroxisome proliferator-activated receptor alpha (PPARα), a nuclear receptor required for lipid homeostasis [20, 21]. As a result, disrupted fatty acid metabolism, including increased omega-9 palmitate-to-oleate ratios, can promote ceramide accumulation, mitochondrial dysfunction, and apoptosis, which contributes to follicular atresia [237].

Based on these observations, supplementation strategies to increase DHEA availability before conception have been proposed, as theca cell synthesis of DHEA occurs approximately 70 days before ovulation. Initiation of supplementation at least four months before planned conception has been recommended to support optimal follicle development [237].

Although research on ovarian aging has traditionally focused on granulosa cells, increasing evidence points to a significant contribution of theca cell dysfunction to age-related decline in female fertility. Strategies that preserve theca cell function and vascular integrity may provide therapeutic opportunities to mitigate reproductive aging.

## Conclusion

In recent years, ovarian theca cells have become recognised as essential components of ovarian biology, acting not only as steroidogenic cells but also as regulators of follicular development, vascularization, immune modulation, and extracellular matrix remodelling. This review outlines the complexity of the theca layer, which contains endocrine cells, fibroblast-like cells, immune cells, vascular elements, and extracellular matrix components that collectively shape a microenvironment required for follicle maturation and ovulation. To our knowledge, this review is the first to integrate recent advances in identification and functional characterization of TSCs, with particular focus on their stem-like properties and proposed roles in follicular vascularization and immune regulation.

Despite these advances, major gaps remain in understanding theca cell biology. The origin, differentiation pathways, and regulatory mechanisms of TSCs remain incompletely defined, particularly in humans. In addition, although theca cell dysfunction is increasingly recognised as a central feature of ovarian pathologies (e.g., PCOS, hyperthecosis, and POI) and ovarian aging, further studies should define the cellular and molecular events that drive these changes. By linking developmental biology, endocrine signaling, and stromal cell interactions, this review provides an integrated perspective and highlights the interconnected functions of follicular cell populations within the ovarian follicle.

Given the fundamental role of theca cells in ovarian physiology, several research directions warrant priority. First, defining the molecular identity of TSCs through specific markers and mapping differentiation trajectories will strengthen regenerative approaches and fertility preservation strategies. Second, clarifying theca–granulosa–oocyte crosstalk by characterizing bidirectional signaling across stages of folliculogenesis should reveal regulatory networks and therapeutic targets. Third, addressing theca cell dysfunction in disorders such as PCOS and POI through metabolic interventions, paracrine modulation, or stem cell-based therapies may improve fertility outcomes. In addition, studies of theca cell aging, including decline in function, vascular integrity, and androgen production, will inform strategies aimed at delaying ovarian aging and extending reproductive lifespan. Finally, defining mechanisms that control theca layer vascularization, given its role in follicular angiogenesis, should support new approaches to preserve or restore ovarian health in both physiological and pathological settings.

These priorities also highlight the translational relevance of TSC biology for regenerative medicine, fertility preservation, and targeted therapies for reproductive disorders such as PCOS and POI.

As critical components of the ovarian niche, theca cells warrant renewed attention as regulators of folliculogenesis and as therapeutic targets in reproductive medicine. A clearer understanding of theca cell biology, including ontogeny, molecular regulation, and intercellular communication, should advance ovarian science and support development of targeted approaches for infertility, ovarian aging, and endocrine disorders.

## Data Availability

No new data were generated or analyzed in support of this research.

## Abbreviations

PCOS: Polycystic ovary syndrome
GCs: Granulosa cells
POI: Primary ovarian insufficiency
BMPs: Bone morphogenetic proteins
TGF-β: Transforming growth factor-beta
VEGF: Vascular endothelial growth factor
ECM: Extracellular matrix
TSCs: Theca stem cells
TPCs: Theca progenitor cells
DHH: Desert hedgehog
IHH: Indian hedgehog
GDF9: Growth differentiation factor 9
MSCs: Mesenchymal stem cells
hTSCs: Human TSCs
MSC: Mesenchymal stem cell
hBMSCs: Human bone marrow mesenchymal stem cells
SCF: Stem cell factor
DHEA: Dehydroepiandrosterone
Evs: Extracellular vesicles
GH: Growth hormone
LH: Luteinizing hormone
IGF1R: IGF1 receptor
FSH: Follicle-stimulating hormone
LIF: Leukemia inhibitory factor
KGF: Keratinocyte growth factor
FGF: Fibroblast growth factor
bFGF: Basic FGF
StAR: Steroidogenic acute regulatory protein
IGF-2: Insulin-like growth factor 2
SCF: Stem cell factor
BMPs: Bone morphogenetic proteins
GDFs: Growth differentiation factors
AMH: Anti-mullerian hormone
CYP17A1: Cytochrome P450 17A1
3β-HSD: 3β-Hydroxysteroid dehydrogenase
CYP17A1: P450 17A1
3β-HSD: 3β-Hydroxysteroid dehydrogenase
LPAR1-4: LPA receptors
PLA2: Phospholipase A2
AX: Autotaxin
BMP-7: Bone morphogenetic protein-7
OCGCs: Oocyte-cumulus-granulosa cell complexes
CTGF: Connective tissue growth factor
FGF-7: Fibroblast growth factor-7
PAI-1: Plasminogen activator inhibitor-1
ROS: Reactive oxygen species
JNK: Jun N-terminal kinase
PCOS: Polycystic ovary syndrome
Esr1: Estrogen receptor alpha gene
INSL3: Insulin-like peptide 3
BM-MSCs: Bone marrow-derived stem cells
